# Prognostic Significance of Tumor Tissue NeuGcGM3 Ganglioside Expression in Patients Receiving Racotumomab Immunotherapy

**DOI:** 10.1155/2020/1360431

**Published:** 2020-06-29

**Authors:** Necdet Uskent, Sule Ayla, Nil Molinas Mandel, Metin Ozkan, Mehmet Teomete, Huseyin Baloglu, Cenk Aydıncer, Hale Yergok, Ender Dogan, Barkın Berk, Aziz Yazar

**Affiliations:** ^1^Department of Medical Oncology, Anadolu Health Center, Istanbul, Kocaeli, Turkey; ^2^Department of Histology and Embryology, School of Medicine, Istanbul Medipol University, Istanbul, Turkey; ^3^Department of Medical Oncology, School of Medicine, Koc University, Istanbul, Turkey; ^4^Department of Medical Oncology, School of Medicine, Erciyes University, Kayseri, Turkey; ^5^Department of Medical Oncology, Altunizade Acibadem Hospital, Istanbul, Turkey; ^6^Department of Pathology, Anadolu Health Science Center, Istanbul, Kocaeli, Turkey; ^7^Department of Pharmaceutical Chemistry, School of Pharmacy, Istanbul Medipol University, Istanbul, Turkey; ^8^Department of Internal Diseases, School of Medicine, Acibadem University, Istanbul, Turkey

## Abstract

Expression of *N*-glycolyl GM3 (NeuGcGM3) ganglioside was detected in the tumor specimens of patients who were on Racotumomab anti-idiotype vaccine maintenance treatment, and prognostic significance as a biomarker was investigated. No statistically significant association was observed in the multivariate analysis between overall survival and tissue NeuGcGM3 IHC levels. Although numerically there was a difference favoring less intense IHC for better prognosis, this did not reach statistical power. However, there was a strong correlation between Racotumomab doses and overall survival (OS). Mean OS of the patient with more than 10 Racotumomab application was significantly longer than the patient who had less than 10 injections (70.7 months vs. 31.1 months, *p* < 0.001). We propose that, regardless of staining intensity, the presence of NeuGcGM3 in patient tissues might be an indicator of benefit in Racotumomab treatment.

## 1. Introduction

Numerous molecules have been considered as targets for cancer immunotherapy because of their levels of expression on tumor cells and their ability of immunogenicity. Among them, a ganglioside GM3 (Neu5Gc) which presents on the outer surface of the plasma membrane of vertebrate cells attracted most attention as a tumor specific antigen, a target for cancer immunotherapy.

**Figure 1:**
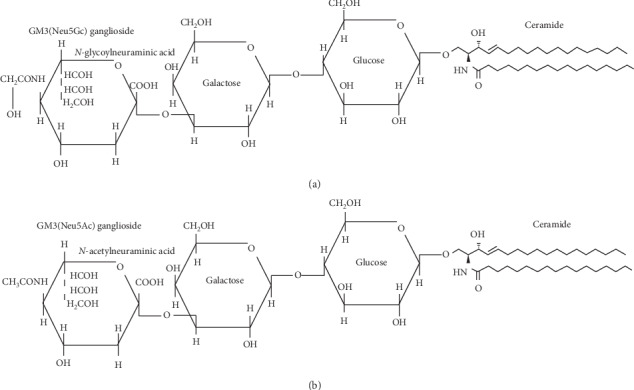
Chemical structures of NeuGcGM3 and NeuAcGM3 [1].

NeuGc is not normally expressed in human cells due to the lack of the enzyme cytidine monophospho-*N*-acetylneuraminic acid hydroxylase, responsible for its synthesis. Aberrant accumulation of NeuGc has been shown in many human malignant cell membranes. In particular, the aberrant expression of the ganglioside NeuGcGM3 in certain human tumors, like lung cancer, breast cancer, neuroblastoma (Scursoni et al. (Clin Dev Immunol, 2011)), pancreatic cancer, and gastrointestinal tumors, made this molecule an attractive target for immunotherapy. The most common sialic acids in humans are *N*-acetyl (NeuAc) and *N*-glycolyl (NeuGc) neuraminic acids(Figure 1) [1–6].

There are numerous hypotheses explaining the existence of NeuGcGM3 in human malignancies, from changing dietary habits to the modified metabolism of malignant cells as a result of genetic mutations and due to the hypoxic conditions of the tumor [7].

Yin et al. showed in their study that hypoxic condition resulted in overexpression of sialic acid transporter sialin and induced cancer-associated gangliosides in human cancer cells [8].

Given its tumor-associated expression, GM3 (Neu5Gc) targeting antibodies have been developed, including the mouse 14F7 monoclonal antibody and its humanized variant 14F7hT [9–11].

Anti-idiotypic antibodies functionally resemble the antigen itself in their own idiotype and Racotumomab is this kind of monoclonal antibody, which mimics NeuGcGM3 and triggers an immune response in various tumors [12–15].

**Figure 2:**
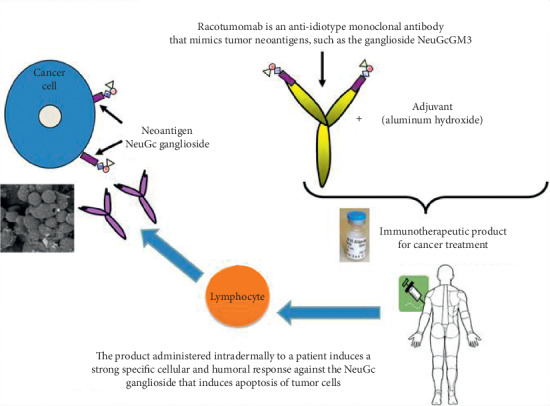
Mechanism of action of Racotumomab [14].

The production of Racotumomab anti-idiotype monoclonal antibody involves the immunization of Balb/c mice with NeuGcGM3 ganglioside, followed by isolation of IgM monoclonal antibody (Ab1). This Ab1 antibody was again used in mice to generate an IgG1 monoclonal antibody (Ab2), named as Racotumomab which has a high affinity towards Ab1 idiotype. Racotumomab by mimicking the ganglioside NeuGcGM3 induces a specific cellular and humoral immune response against the actual antigen (Figure 2).

Segatori et al. demonstrated that anti-NeuGcGM3 Abs induced by Racotumomab vaccination are able to mediate an antigen-specific antibody-dependent cellular cytotoxicity (ADCC) response against tumor cells in NSCLC patients [16].

Patients vaccinated with Racotumomab produce antibodies against NeuGcGM3. Hernández et al. demonstrated that immunized patients' sera had cytotoxic effects on tumor cells in the L1210 culture expressing NeuGcGM3 [17].

Racotumomab is currently registered in Cuba and Argentina in non-small-cell lung cancer (NSCLC) as a second-line treatment. In a multicenter double-blind randomized, placebo controlled Phase II/III trial involving 176 patients with advanced NSCLC, Alfonso et al. demonstrated an overall survival benefit, almost doubled in one year (22.5% vs. 40.2%) and tripled in 2 years (6.7% vs. 18.4%) favoring the study group [18].

The prognostic role of NeuGcGM3 expression in NSCLC is controversial. Blanco et al. assessed the relationship between NeuGcGM3 expression intensity and overall survival of advanced NSCLC patients using 14F7 Mab and concluded that NeuGcGM3 expression was associated with higher proliferation index and poor OS of patients. In their study, five-year survival probability in NSCLC patients with higher levels of NeuGcGM3 expression was 3 to 4 times lower than those with reduced levels [3].

In another study, a group from Japan confirmed the high expression of NeuGc gangliosides in NSCLC using the monoclonal antibody GMR8, which is specific to gangliosides with NeuGc alpha 2,3Gal-terminal structures and demonstrated for the first time that an inverse relationship exists between NeuGcGM3 expression levels and progression-free survival [19].

There are also other studies claiming that higher tissue NeuGcGM3 levels represent a more aggressive phenotype, like EGFR and EGF expressions [19, 20].

### 1.1. Aim of the Study

Although scarce reports in the literature suggested that high tissue NeuGcGM3 levels might represent a more aggressive tumor, prognostic implication of this was not known in the patients who are on anti-NeuGcGM3 treatments. In this study, we investigated the prognostic significance of tissue NeuGcGM3 expression level in the patients who are on switch maintenance treatment with Racotumomab.

## 2. Materials and Methods

This study was accredited by Noninvasive Clinical Studies Ethical Committee of Istanbul Medipol University, registration number 10840098-604.01.01-E.44203. Expression of NeuGcGM3 was detected in the tumor specimens of 25 patients, using 14F7 monoclonal antibody (14F7 Mab) by immunohistochemistry (IHC) test and graded as 0, 1+, 2+, or 3+ according to the intensity of the stain. IHC staining was also performed in 10 benign tissue specimens as controls. 14F7 monoclonal antibody was obtained from mice that were immunized with purified gangliosides and supplied by the manufacturer's institute, Center of Molecular Immunology (CIM), Havana, Cuba.

Detection of the ganglioside in the tumor tissue was done by IHC with primer antibody: 14F7 Mab 20 mcgr/ml after using biotinylated link universal and a streptavidin HRP commercial kit. Afterwards, the enzymatic activity was visualized with a diaminobenzidine (DAB). All samples were counterstained with Mayer's Hematoxylin. Immunoreactivity on the sample was evidenced as a brown staining. 5-microns thick sections of tissue (paraffin-embedded block) were used to observe the expression of NeuGcGM3.

IHC using 14F7 Mab was evaluated according to the percentage of positive cells (0–100 percent) and graded as 0, 1+ (weak), 2+ (moderate), or 3+ (strong) according to the intensity of the reaction. The score was calculated for each specimen by multiplication of the intensity of reaction and the grade of positive cells. Classification of the score was slightly modified from the method used by Blanco et al. as follows: 0 (total score 0); IHC 1+ (total score 1–100); IHC 2+ (total score 101–150); and IHC 3+ (total score > 150) [3, 15].

### 2.1. Racotumomab Administration

The route of administration was intradermal; each dose was divided into four different anatomic sites in the medial surfaces of arms, thighs, or abdomen. The vaccination program consisted of two phases. In the induction phase, 5 doses were administered 2 weeks apart, followed by the maintenance phase, when 10 more doses were administered monthly.

## 3. Results

All 48 patients were on Racotumomab maintenance treatment following chemotherapy who had at least partial remission. Out of a total of 48 patients, 42 had lung cancer (40 NSCLC, 2 SCLC), 6 had miscellaneous cancers (breast, pancreas, etc.) of 40 non-small-cell lung cancer (NSCLC) patients, 12 patients were in stage III, and 28 were in stage IV at the time of the diagnoses. 19 adenocarcinomas and 21 squamous cell carcinomas were identified. None of the patients had sensitive mutations like EGFR, ALK, and ROS-1. There were also 2 patients with small-cell lung cancer (SCLC). Male/female ratio was 3.36 (37/11) and median age was 63 (31–85) (Table 1).

**Figure 3:**
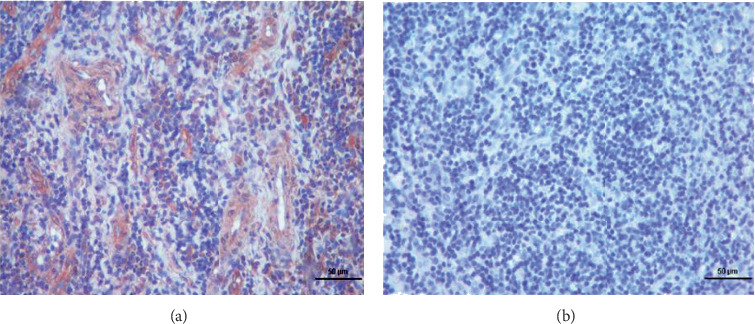
Positive and negative IHC staining results for NeuGcGM3. (a) Positive IHC 2+ staining for GM3, X10. (b) Negative IHC result for GM3, X10.

NeuGcGM3 IHC was performed in 25 out of 48 patients whose paraffin blocks were available and adequate for testing. 9 patients had strong 3+, 12 had moderate 2+, and 4 patients had weak 1+ NeuGcGM3 staining intensity. None of the benign tissues, as controls, showed positivity. Positive IHC staining of NeuGcGM3 from a patient's tumor tissue with NSCLC and a control staining of a benign tissue using 14F7 Mab are depicted in Figure 3.

Mean overall survival (OS) time for subsequent IHC groups was 88.1 months for IHC 1+, 51.6 months for IHC 2+, and 51.7 months for IHC 3+. Mean survival of the patients who died was 22.6 months for IHC 3+ and 27.9 months for IHC +2, whereas in living patients, mean OS was 51.1 months in IHC 3+, compared to 57.8 months and 74.3 months in IHC 2+ and IHC 1+ patients, consecutively.

**Figure 4:**
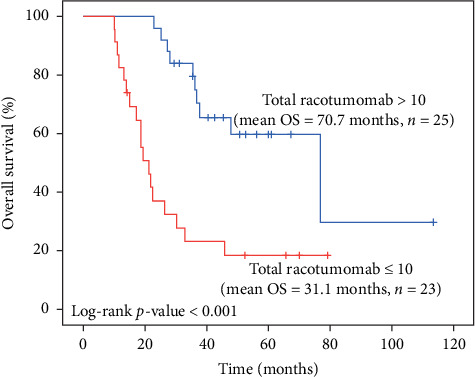
Kaplan–Meier survival estimates by the number of total Racotumomab cycles used during the course of treatment (*n* = 48).

In the multivariate Cox regression model, all the variables in Table 2 were included in the model to identify the independent predictors of survival. The analysis showed that the total number of Racotumomab cycles used during the course of treatment was statistically associated with better overall survival rates (HR = 0.750, 95% CI 0.601–0.936, *p*=0.011), as was also found in the univariate analysis in Figure 4. That is, after adjusting for age, gender, IHC, and treatment protocol, important reduction in mortality rate was attributed to each dose of Racotumomab.

The total number of Racotumomab cycles used in the course of treatment was categorized into two groups as ≤10 (*n* = 23) and >10 (*n* = 25), and it was found that more than 10 cycles significantly were associated with better overall survival (*p* < 0.001) (Figure 4).

When IHC groups were considered, the patients with IHC scores of 2 have 2.6 times higher risk of death compared to the patients with IHC scores of 1. Also, the patients with IHC scores of 3 have 1.3 times higher risk of death compared to the patients with IHC scores of 1. Due to the small sample size, the findings regarding the IHC groups were not found to be statistically significant (Table 2).

Univariate (Table 3) and multivariate (Table 2) survival analyses were conducted to compare overall survival (OS) rates. Survival curves were generated with the Kaplan–Meier method and log-rank test was used to compare survival curves as the univariate survival analysis. Multivariate analysis was performed with Cox proportional hazards regression to identify the independent risk factors of OS.

**Figure 5:**
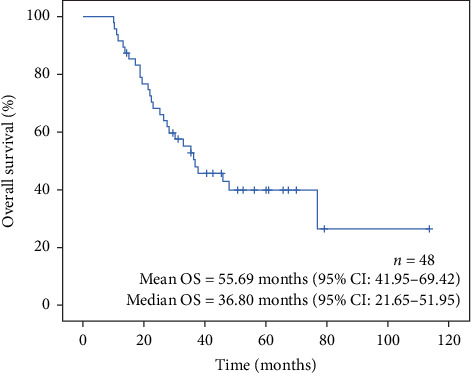
Overall survival curve for all patients (*n* = 48).

Median follow-up time was 34.2 months, ranging from 10.1 to 113.7 months. After 5 years of follow-up, 27/48 (56.25%) of patients died and 11/48 (22.9%) were alive more than 5 years after diagnosis (Figure 5).

**Figure 6:**
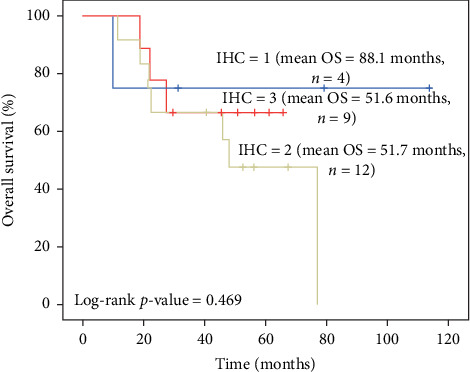
Kaplan–Meier survival estimates by IHC scores.

The overall survival rate throughout the follow-up period was found to be higher in the patients with IHC score of 1, with mean overall survival of 88.1 months (95% CI: 43.9–132.2). When the patients with IHC scores of 2 and 3 were considered, the mean of overall survival time was approximately the same (51.7 months (95% CI: 35.5–67.9) vs. 51.6 months (95% CI: 38.14–64.97), respectively). However, the sample size was limited to draw any statistically significant difference between IHC groups (Figure 6).

## 4. Discussion

Pilco-Jeneto and colleagues assessed the expression of NeuGcGM3 in fifty paraffin embedded specimens from patients diagnosed with sarcomas and investigated the relation of this expression with clinicopathological features and overall survival of patients. They concluded that expression of NeuGcGM3 was a significantly poor prognostic factor for overall survival [19].

Blanco et al. investigated the relationship between the NeuGcGM3 expression, Epidermal Growth Factor Receptor (EGFR), and Epidermal Growth Factor (EGF) with overall survival of patients with advanced NSCLC. Higher NeuGcGM3 expression was associated with a poorer OS of the patients. Moreover, the double and triple positivity of tumors with EGFR and EGF identified more aggressive biological behavior in both univariate (*p* : 0.020) and multivariate analyses (*p* : 0.010) [3].

In their work, Blanco et al. evaluated the expression of NeuGcGM3 using 14F7 Mab and proposed an IHC score called H-score. The authors classified the staining intensity as 0 (0), 1 (1 to 100), 2 (101–200), and 3 (>200). However, subsequently, they divided H-scores into 2 group as low expression (scores < 150) and high expression (scores > 150), while in our study we decided to use a slightly different scoring system like 0 (0), 1 (1–100), 2 (101–150), and 3 (>150). This modification was proposed by our Pathology Department due to the scarcity of that >200 staining.

NeuGcGM3 is capable to bind to the extracellular domain of the epidermal growth factor receptor (EGFR). This binding may provoke EGFR system activation mediated by the growth factor ligand. Activation of epidermal growth factor in the presence of overexpressed NeuGcGM3 constitutes a more aggressive immunophenotype [3]. In our oncoming studies, we are planning to include tissue EGFR levels and serum EGF levels in addition to tissue NeuGcGM3 expression, in order to better understand the value of anti-EGF plus anti-NeuGcGM3 combination strategies.

To the best of our knowledge, there were no studies in the literature, assessing the predictive and prognostic value of NeuGcGM3 expression in the tumor tissues who are on anti-NeuGcGM3 antibody treatment. In our study, we demonstrated a strong relation with Racotumomab dose cycles and overall survival of the patients. Mean survival of the patient who received more than 10 intradermal injections was significantly higher than the patient who received less than 10 injections (70.7 months vs. 31.1 months, *p* < 0.001).

In the multivariate Cox regression model, all the variables were included in the model to identify the independent predictors of survival (Table 2). The analysis showed that the total number of Racotumomab cycles used during the course of treatment was statistically associated with better overall survival rates (HR = 0.750, 95% CI 0.601–0.936, *p*=0.011). This finding was also confirmed in the univariate analysis; that is, after adjusting for age, gender, IHC, and treatment protocol, the 25% reduction in mortality rate was attributed to the cumulative dose of Racotumomab (log-rank *p* value <0.001).

These findings may be well comparable with HER-2 expression in breast cancer. HER-2 was considered a bad prognostic biomarker, a messenger for progressive disease before anti-HER2 strategies had been invented. However, today, best outcomes are achieved in this subtype.

The presence of NeuGcGM3 antigen is favorable for the treatment with Racotumomab since its antitumor activity is mediated by an immune response directed against the NeuGcGM3 antigen. Thus, we may argue that regardless of the intensity of the staining, the presence of NeuGcGM3 at the tissues of patients is an indicator of benefit in Racotumomab treatment.

Our study did not show any significant association between tissue antigen staining intensity and overall survival, probably due to the small number of patients that were analyzed. However, this may also be the result of compensatory effect of Racotumomab, balancing aggressive biological phenotype with anti-NeuGcGM3 effect.

In our previous research, we investigated the prognostic significance of serum anti-NeuGcGM3 antibodies and cytotoxicity tests in cell lines using hyperimmune patient's sera of the patients who are on Racotumomab treatment. In that study, we concluded that cytotoxicity test was a good tool as a biomarker of response to Racotumomab immunotherapy, also related to the better prognosis of the patients with positive tests. However, in the same study, tissue NeuGcGM3 levels did not give us valuable information as to the prediction of response or prognosis although the number of the patients was limited [21].

## 5. Conclusion

No statistically significant association was observed in the multivariate analysis between overall survival and tissue NeuGcM3 IHC level. Although numerically there was a difference favoring less intense IHC for better prognosis, this did not reach statistical power.

However, looking at the overall survival curve of our patients, we concluded that, regardless of the intensity of staining, the presence of NeuGcGM3 at the tissues of patients is an indicator of benefit in Racotumomab treatment. We believe although high expression of NeuGcGM3 of tumor tissues is generally considered as a poor prognostic marker, anti-NeuGcGM3 treatment with Racotumomab overrides this phenomenon.

## Figures and Tables

**Table 1:** Patients demography on Racotumomab treatment.

	Total patients (*n*)	NSCLC adeno	NSCLC sq cell	SCLC	Miscel.
Number of patients	48	19	21	2	6
Female	11	6	2	1	2
Male	37	13	19	1	4
Mean age	63.0	62.4	60.2	51.6	58.1
Stage					—
IV	28^*∗*^	13	15	2	6
IIIB	10^*∗*^	5	5	—	—
IIIA	2^*∗*^	1	1	—	—

NSCLC: non-small-cell lung cancer; adeno: adenocarcinoma; sq cell: squamous cell; Miscel.: miscellaneous. ^*∗*^Patients with NSCLC (adeno and sq cell) in different stages.

**Table 2:** Multivariate analysis of overall survival with Cox proportional hazards regression analysis.

Variables	HR (95% CI)	*p* value
Age	0.940 (0.869–1.016)	0.119
Gender, male	3.920 (0.290–52.896)	0.304
IHC		
+1	Reference	
+2	2.615 (0.156–43.928)	0.504
+3	1.311 (0.052–33.028)	0.869
Chemotherapy + Racotumomab treatment	0.298 (0.038–2.316)	0.247
Total Racotumomab	0.750 (0.601–0.936)	**0.011**

IHC: immunohistochemistry; HR: hazard ratio; CI: confidence interval. Bold *p* value indicates statistically significant association.

**Table 3:** Univariate comparisons between patients that were alive and dead.

	Alive (*n* = 20)	Dead (*n* = 28)
Age, years (mean ± SS)	63.1 ± 11.5	63.8 ± 11.1
Gender		
Male	14	23
Female	6	5
IHC		
+3	6	3
+2	5	7
+1	3	1
Not available	6	17
Total Racotumomab (mean ± *SS*)	13.9 ± 5.2	9.6 ± 4.0

Fisher's exact test and independent samples *t*-test were used for categorical and continuous variables, respectively.

## Data Availability

The data provided in this publication will be available from the corresponding author upon request.

## References

[1] Labrada M., Dornvignit D., Hevia G. (2018). GM3 (Neu5Cc) ganglioside: an evolution fixed neoantigen for cancer immunotherapy. *Seminars in Oncology*.

[2] Scursoni A. M., Galluzzo L., Camarero S. (2011). Detection of *N*-glycolyl GM3 ganglioside in neuroectodermal tumors by immunohistochemistry: an attractive vaccine target for aggressive pediatric cancer. *Clinical and Developmental Immunology*.

[3] Blanco R., Domínguez E., Morales O. (2015). Prognostic significance of *N*-glycol GM3 ganglioside expression in non-small cell lung carsinoma patients: new evidences. *Pathology Research International*.

[4] Blanco R. (2016). *N*-glycolyl GM3 ganglioside as a relevant tumor antigen in humans. *Journal of Molecular Biomarkers & Diagnosis*.

[5] Carr A., Mullet A., Mazorra Z. (2000). A mouse IgG1 monoclonal antibody specific for *N*-glycolyl GM3 ganglioside recognized breast and melanoma tumors. *Hybridoma*.

[6] Blanco R., Rengifo E., Cedeño M., Rengifo C. E., Alonso D. F., Carr A. (2011). Immunoreactivity of the 14F7 mab raised against *N*-glycolyl GM3 ganglioside in epithelial malignant tumors from digestive system. *ISRN Gastroenterology*.

[7] Tangvoranuntakul P., Gagneux P., Diaz S. (2003). Human uptake and incorporation of an immunogenic nonhuman dietary sialic acid. *Proceedings of the National Academy of Sciences*.

[8] Yin J., Hashimoto A., Izawa M. (2006). Hypoxic culture induces expression of sialin, a sialic acid transporter, and cancer-associated gangliosides containing non-human sialic acid on human cancer cells. *Cancer Research*.

[9] Blanco R., Quintena Y., Blanco D. (2013). Tissue reactivity of the 14F7 mab raised against *N*-glycolyl GM3 ganglioside in tumors of neuroectodermal, mesodermal and epithelial origin. *Journal of Biomarkers*.

[10] Dorvignit D., Boligan K. F., Relova-Hernández E. (2019). Antitumor effects of GM3 ganglioside-specific humanized antibody 14F7hT against Cmah-transfected cancer cells. *Scientific Reports*.

[11] Dorvignit D., García-Martínez L., Rossin A. (2015). Antitumor and cytotoxic properties of a humanized antibody specific for the GM3 (Neu5Gc) ganglioside. *Immunobiology*.

[12] Alfonso M., Díaz A., Hernández A. M. (2002). An anti-idiotype vaccine elicits a specific response to *N*-glycolyl sialic acid residues of glycoconjugates in melanoma patients. *The Journal of Immunology*.

[13] Alfonso S., Díaz R. M., De La Torre A. (2007). 1E10 anti-idiotype vaccine in non-small cell lung cancer: experience in stage IIIb/IV patients. *Cancer Biology & Therapy*.

[14] Gomez R. E., Ardigo M. L. (2012). Anti-idiotype antibodies in cancer treatment: the pharmaceutical industry perspective. *Frontiers in Oncology*.

[15] Blanco R., Rengifo C. E., Cedeño M., Frómeta M., Rengifo E., Carr A. (2012). Immunoreactivity of the 14F7 mab (raised against *N*-glycolyl GM3 ganglioside) as a positive prognostic factor in non-small-cell lung cancer. *Pathology Research International*.

[16] Segatori V. I., Cuello H. A., Gulino C. A. (2018). Antibody-dependent cell-mediated cytotoxicity induced by active immunotherapy based on racotumomab in non-small cell lung cancer patients. *Cancer Immunology, Immunotherapy*.

[17] Hernández A. M., Rodríguez N., González J. E. (2011). Anti-NeuGcGM3 antibodies, actively elicited by idiotypic vaccination in nonsmall cell lung cancer patients, induce tumor cell death by an oncosis-like mechanism. *The Journal of Immunology*.

[18] Alfonso S., Valdes-Zayas A., Santiesteban E. R. (2014). A randomized, multicenter, placebo-controlled clinical trial of racotumomab-alum vaccine as switch maintenance therapy in advanced non-small cell lung cancer patients. *Clinical Cancer Research*.

[19] Hyashi N., Chiba H., Kuronumo K. (2013). Detection of *N*-glycosylated gangliosides in non-small cell lung cancer using GMR8 monoclonal antibody. *Cancer Science*.

[20] Pilco-Janeta D., De la Cruz Puebla M., Soriano J. (2019). Aberrant expression of *N*-glycolyl GM3 ganglioside is associated with the aggressive biological behavior of human sarcomas. *BMC Cancer*.

[21] Uskent N., Mandel N. M., Gulbas Z. (2017). Cytolytic tests with hyperimmune patient sera is a good prognostic tool in racotumomab immunotherapy in advanced non-small cell lung cancer. *Advances in Modern Oncology Research*.

